# A novel sunflower broomrape race with unusual virulence potentially caused by a mutation

**DOI:** 10.3389/fpls.2023.1236511

**Published:** 2023-10-06

**Authors:** Belén Fernández-Melero, Alberto Martín-Sanz, Lidia del Moral, Begoña Pérez-Vich, Leonardo Velasco

**Affiliations:** ^1^ Department of Plant Breeding, Instituto de Agricultura Sostenible – Consejo Superior de Investigaciones Científicas (IAS-CSIC), Córdoba, Spain; ^2^ Syngenta España S.A., Carmona, Spain

**Keywords:** intrapopulation diversity, *Orobanche cumana*, population genetics, racial evolution, virulence

## Abstract

**Introduction:**

The sunflower broomrape (*Orobanche cumana* Wallr.) gene pools of the Guadalquivir Valley and Cuenca province in Spain had predominantly race-F virulence. A new race G was observed recently in the Guadalquivir Valley potentially due to the genetic recombination of the avirulence genes of both gene pools.

**Methods:**

In this research, we have studied populations with atypical virulence from Cuenca. These populations parasitize on DEB2 sunflower line, resistant to all race-G populations evaluated. Ten populations collected in Cuenca province were evaluated with sunflower differential lines and genotyped with 67 SNP markers.

**Results:**

Although genetic recombination with individuals of the Guadalquivir Valley gene pool has been observed in most populations, recombination of avirulence genes was discarded as the cause of the new virulence because the population with the highest degree of attack on DEB2 showed no introgression from an external gene pool. Accordingly, a point mutation is proposed as the putative cause of the new virulence.

**Discussion:**

The present study provided a detailed characterization of each population, including the accurate classification of the individuals belonging to each of the classical Spanish gene pools, F1 hybrids, and those that evolved from hybridization between both gene pools. This information is essential to understand how sunflower broomrape populations are evolving in Spain, which in turn may be helpful to understand the dynamics of sunflower broomrape populations in other areas of the world and use this information to develop durable strategies for resistance breeding.

## Introduction

The holoparasitic plant species sunflower broomrape (*Orobanche cumana* Wallr.) is currently one of the main constraints for sunflower production in most sunflower-producing areas. This species is currently distributed across Europe and Asia (Fernández-Martínez et al., 2015) and is spreading to other areas, such as Africa (Amri et al., 2012; Nabloussi et al., 2018). Sunflower yield losses due to broomrape infestation can surpass 50% when susceptible hybrids are grown, reaching even 100% under conditions of heavy infestation (Cvejić et al., 2020). In the wild, sunflower broomrape has a narrow range of host plants restricted to the Asteraceae family, particularly species of Artemisia, across a geographic range stretching from Central Asia to South-eastern Europe (Pujadas-Salvà and Velasco, 2000). In agricultural production, sunflower broomrape is host-specific and only parasitizes on sunflower crops (Fernández-Martínez et al., 2015).

Sunflower broomrape produces numerous minuscule seeds that remain in a dormant state within the soil until their germination is triggered by specific compounds exuded by the host plant, primarily strigolactones and sesquiterpene lactones (Raupp and Spring, 2013). Upon germination, the germ tube elongates its cells towards the host plant’s roots. When it reaches them, its apical cells differentiate into papillae secreting a mucilaginous substance that promotes adhesion to the host. After attachment, the germ tube produces a swollen structure at the apex, the prehaustorium, whose intrusive cells progress through the host root tissues (Delavault, 2015). Once the pre-haustorium penetrates the endodermis, parasite cells progress toward host vessels and then initiate a specialized endophytic organ, the haustorium *sensu stricto*, that proceeds to the establishment of vascular connections and enables the parasite to uptake water and nutrients from the host conductive system (Delavault, 2015). Then, broomrape begins to derive phloemic flow acting as a strong nutrient sink and a nutrient storage organ called tubercle develops quickly from which an underground shoot develops (Krupp et al., 2019). The shoots emerge from the soil, flower, and produce new seeds that contribute to the expansion of the seed bank (Molinero-Ruiz et al., 2015).

In contrast to most parasitic systems involving *Orobanche* or the closely related *Phelipanche* species, the interaction between sunflower broomrape and sunflower is primarily characterized by a gene-for-gene relationship. In this interaction, a dominant resistance gene in the sunflower host interacts with a corresponding dominant avirulence gene in the broomrape parasite (Rodríguez-Ojeda et al., 2013). This type of interaction, characterized by vertical or qualitative resistance mechanisms within the host, gives rise to distinct physiological races of the parasite (Fernández-Martínez et al., 2015). These races are designated with letters, starting with A, representing the initial race observed in Russia during the nineteenth century, and extending to G, which is currently the most virulent race prevalent in many regions (Cvejić et al., 2020). Nevertheless, it remains uncertain whether populations labeled with the same letter in various parts of the world truly represent the same physiological race, given the absence of a universally applicable set of differential lines for race classification (Velasco et al., 2016). For that reason, Martín-Sanz et al. (2016) proposed adding a subscript abbreviation to the letter of the race to indicate the country or region of origin.

Broomrape race evolution has been parallel in most of the areas where the parasite has been present for a long time, probably because the resistance genes deployed in commercial cultivars were the same. In these areas, race E was the most virulent race at the end of the 1970s (Vranceanu et al., 1980). Populations with increased virulence, named race F were detected in 1995 in Spain (Alonso et al., 1996) and shortly after in most areas of Eastern Europe (Škorić et al., 2010). New populations of race G overcoming race-F-resistant hybrids were first identified in Turkey (Kaya et al., 2004) and not long after in the main sunflower-producing areas of Europe and Asia (Kaya, 2014).

In Spain, where sunflower broomrape is not present in the wild flora, and the parasite only occurs in agricultural fields after its introduction from other areas, two genetically separated gene pools have been identified, one of them in the Guadalquivir Valley in southern Spain and another in Cuenca province in central Spain. Both gene pools exhibit shallow genetic diversity due to founder effects (Pineda-Martos et al., 2013). These authors observed for the first time the admixture of plants from both gene pools in a few sunflower fields. They also documented the existence of cross-fertilization between individuals of both gene pools in some of the populations, although genetic recombination did not affect virulence. A few years later, Martín-Sanz et al. (2016) identified populations in southern Spain derived from the genetic recombination of the Guadalquivir and Cuenca gene pools that possessed increased virulence compared to the original race-F populations. These populations overcame the genetic resistance in most race-F-resistant hybrids tested and were classified as race G_GV_. The authors hypothesized that increased virulence resulted from the genetic recombination of avirulence genes from the two gene pools. It is important to recall that sunflower and sunflower broomrape generally follow a gene-for-gene interaction, in which a resistance gene in the crop interacts with an avirulence gene in the parasite (Vranceanu et al., 1980; Rodríguez-Ojeda et al., 2013).

The situation of the Cuenca gene pool has remained unexplored since the study of Pineda-Martos et al. (2013). Meanwhile, it was noticed that some populations of that area possessed the ability to parasitize on DEB2 sunflower line, which is resistant to race-G populations from eastern Europe and southern Spain (Martín-Sanz et al., 2016). Accordingly, this study aimed to evaluate the virulence and population structure of a set of sunflower broomrape populations collected in Cuenca Province, Spain.

## Materials and methods

### Sunflower broomrape populations

The populations used in the study are listed in **Table 1**. They include 10 populations collected in Cuenca province and characterized for the first time in this research. Seed collection from these populations was conducted in September 2014 and 2020 on commercial sunflower fields, in all cases on a single cultivar. The level of broomrape resistance of the cultivar is indicated in **Table 1** in the cases in which it was known. Mature broomrape plants growing on at least 50 sunflower plants were cut and placed in paper bags. Individual paper bags were labeled, folded, and stapled to avoid seed contamination during transportation. Additionally, the following populations were used: two populations of race F from the Guadalquivir Valley (F_GV_), two populations of race F from Cuenca (F_CU_), and three populations of race G from the Guadalquivir Valley (G_GV_). These populations were used as controls in the molecular studies to characterize population structure and genetic diversity. In the experiments for evaluating virulence against a set of differential lines, only the G_GV_ populations were used as controls. The use of F_GV_ populations as controls in the study of virulence was considered unnecessary since they have lower virulence than G_GV_ populations (Martín-Sanz et al., 2016). No viable seeds of the F_CU_ gene pool from populations that can be unequivocally considered pure were available.

**Table 1:** Data of collection and role of the sunflower broomrape populations used.

Population	Location	Province	Date	Sunflower type	Role	Race
NCU1	Palomares del Campo	Cuenca	2014	Race-F resistant	Under study	
NCU2	Palomares del Campo	Cuenca	2020	Unknown	Under study	
NCU3	Palomares del Campo	Cuenca	2020	Unknown	Under study	
NCU4	Palomares del Campo	Cuenca	2020	Race-F resistant	Under study	
NCU5	Montalbo	Cuenca	2020	Race-E resistant	Under study	
NCU6	Montalbo	Cuenca	2020	Race-E resistant	Under study	
NCU7	Torralba	Cuenca	2020	Race-E resistant	Under study	
NCU8	Cuenca	Cuenca	2020	Unknown	Under study	
NCU9	Naharros	Cuenca	2014	Unknown	Under study	
NCU10	La Almarcha	Cuenca	2014	Race-F resistant	Under study	
SE10	Écija	Sevilla	2007	Race-E resistant	Control for molecular studies	F_GV_
CO02	Aldea Quintana	Córdoba	1995	Unknown	Control for molecular studies	F_GV_
CU05	La Almarcha	Cuenca	1996	Unknown	Control for molecular studies	F_CU_
CU08	Carrascosa del Campo	Cuenca	1996	Unknown	Control for molecular studies	F_CU_
NGV1	Las Cabezas	Sevilla	2014	Race-F resistant	Control for molecular and virulence studies	G_GV_
NGV2	Écija	Sevilla	2014	Race-F resistant	Control for molecular and virulence studies	G_GV_
NGV3	Marchena	Sevilla	2014	Race-F resistant	Control for molecular and virulence studies	G_GV_

### Sunflower differential lines

Sunflower lines used to test the virulence of the broomrape populations were: B117, susceptible to all known broomrape races; NR5, which has the *Or5* alleles that confer resistance to race E; P96, with oligogenic resistance to race F (Fernández-Martínez et al., 2004); Kiara, which is a representative commercial hybrid with race-F resistance; LP2, an inbred line containing *Or7* alleles conferring resistance to race F, developed by the authors for research purposes from a commercial hybrid; and DEB2, which contains the *Or_Deb2_* gene that confers resistance to race-G populations from eastern Europe and southern Spain (Fernández-Aparicio et al., 2022). In all these lines, the resistance is cumulative, i.e., the line resistant to race G is also resistant to previous races such as F and E.

### Evaluation of virulence

Tests to evaluate the virulence of broomrape populations against the set of differential lines were conducted in the greenhouse in winter-spring of 2021-2022 and under open-air conditions in spring-summer of 2022, in both cases using eight 6-L pots per combination of sunflower line and broomrape population, each one containing a single plant. All the sunflower plants were inoculated artificially by adding around 30 mg of broomrape seeds to small pots (7 x 7 x 7 cm) filled with sand and peat in a proportion of 1:1 by volume, shaking the mixture vigorously in a plastic bag. Then, previously germinated sunflower seeds were planted in the pots and maintained in a growth chamber for four weeks at 25°C/20 °C (day/night) and a photoperiod of 16 h light: 8 h dark. After this, the plants were transplanted into the 6-L pots filled with sand, silt, and peat in a proportion of 2:1:1 by volume. The evaluation was conducted by counting the number of emerged *O. cumana* shoots per sunflower plant at sunflower maturity.

Data were analyzed through ANOVA with the environment (greenhouse, open-air), sunflower line, and broomrape population as fixed factors. To compare the performance of broomrape populations on each of the sunflower differential lines, ANOVA was repeated for each of the differential lines separately and Tukey’s *post hoc* test for multiple mean comparisons was computed.

### Plant genotyping

Apical tissue from 12 to 40 broomrape shoots parasitizing the susceptible line B117 was collected for the populations studied and the controls. In the case of control lines SE10, CO02, CU05, and CO08, DNA was extracted from 12 plants per population in 2015. For NCU1, for which we had previous evidence of shallow intrapopulation diversity and atypical virulence, DNA was extracted from 40 individual plants. For the other populations, DNA was extracted from 24 plants per population. Apical tissue of young broomrape shoots was collected and stored at -80 °C. The tissue of individual shoots was then freeze-dried and ground in a laboratory ball mill. DNA was extraction was done following the procedure described by Rogers and Bendich (1985) with the following adaptations: a) addition of 0.1% (w/v) ascorbic acid, 0.1% (w/v) diethyldithiocarbamic acid sodium salt, and 0.2% (v/v) 2-mercaptoethanol to the CTAB extraction buffer; b) CTAB buffer incubation time of 30 min; and c) use of chloroform instead of chloroform: isoamyl alcohol 24:1. Genotyping of individual broomrape plants was conducted with 67 highly polymorphic SNP markers selected from the 192 *O. cumana* SNP marker set reported and mapped by Calderón-González et al. (2019). KASP genotyping assays were conducted at LGC Biosearch Technologies, Teddington, Middlesex, UK.

### Genetic diversity and population structure analyses

Genetic diversity within each population and genetic distances between populations were analyzed using GenAlEx ver. 6.5 (Peakall and Smouse, 2012). The following parameters of intrapopulation diversity were calculated: the percentage of polymorphic loci (P), the observed heterozygosity (Ho), the expected (He) heterozygosity, and Shannon’s diversity index (I). Nei’s unbiased genetic identity between populations was also computed. Seven individuals with >10% missing data were excluded from the analysis. Raw data are provided in **Supplementary Table 1**.

A principal coordinates analysis (PCoA) was conducted from the matrix of GST pairwise distances to have an overview of the structure of the populations and their relatedness. Output graphics were prepared using a combined set of the population groups (race F_GV_, race G_GV_, F_CU_, and the populations from Cuenca under study) and, afterward, the individual populations, excluding the race G_GV_ populations, to simplify the graph.

Genetic structure analysis was conducted using STRUCTURE ver. 2.3.4 using a clustering method with admixture (Pritchard et al., 2000). The analysis considered between one and ten expected populations (K) and was repeated ten times for each value of K. The number of Markov chain Monte Carlo (MCMC) iterations was set to 100,000 using a burning period of 10,000. The expected number of clusters in the data set was estimated using Structure Harvester (Earl and Von Holdt, 2012). Cluster membership of the individuals included in the analysis was resolved using the FullSearch algorithm of CLUMPP ver. 1.1.2b (Jakobsson and Rosenberg, 2007), and the output was used to produce bar graphs of the population structure using OriginPro 2022b software (OriginLab Corporation, Northampton, MA, USA). Since the results suggested the existence of two genetic groups, putative F_1_ individuals were defined as those having a membership to each group between 49.90% and 50.10%.

## Results

### Virulence of the populations

The analysis of variance revealed statistical significance (*P*<0.01) of the sunflower line, the broomrape population, and all the interactions, but not of the environment (greenhouse vs open-air conditions; *P*=0.16). The analyses conducted on the individual sunflower differential lines showed that all broomrape populations infected severely the susceptible line B117 and the race-E resistant line NR5, which pointed out that they belong to race F or above. The main differences between the NCU populations were observed in their reactions on the differential genotypes Kiara and DEB2. Populations NC1 to NCU8 did not parasitize on Kiara or showed a very low number of emerged shoots per plant. Populations NCU9 and NCU10 showed a higher number of shoots per plant, although in the case of NCU10 the difference was not significant. The three race-G_GV_ populations used as a control showed parasitization on Kiara. The reaction was inverse in the case of the race-G resistant line DEB2: populations NCU9 and NCU10 exhibited a complete absence of parasitization on DEB2, whereas populations NC1 to NCU8 showed some degree of parasitization on this line, which was statistically significant only for NCU1 to NCU4. Parasitization on the race-F resistant lines P96 and LP2 was null or very low in all cases (**Table 2**).

**Table 2:** Number of emerged broomrape shoots per plant parasitizing the sunflower differential lines B117 (no resistance), NR5 (resistance to race E, gene *Or_5_*), Kiara (commercial hybrid resistant to race F), P96 (oligogenic resistance to race F), LP2 (resistance to race F, gene *Or_7_*), and DEB2 (resistance to race G, gene *Or_Deb2_*) using ten sunflower broomrape populations from Cuenca province (Central Spain) and three race-G populations from the Guadalquivir Valley in Sothern Spain, the latter used as controls^a^.

O. cumana	B117	NR5	Kiara	P96	LP2	DEB2
NCU1	12.56 ± 5.35^abc^	16.94 ± 5.18^bcd^	0.00 ± 0.00^a^	0.00 ± 0.00^a^	0.00 ± 0.00^a^	12.44 ± 5.16^e^
NCU2	10.63 ± 5.73^ab^	18.50 ± 3.93^cd^	0.00 ± 0.00^a^	0.63 ± 1.26^ab^	0.00 ± 0.00^a^	7.88 ± 5.97^d^
NCU3	9.00 ± 4.69^a^	15.56 ± 2.76^bcd^	0.63 ± 2.50^a^	0.63 ± 1.26^ab^	0.00 ± 0.00^a^	4.75 ± 4.23^bc^
NCU4	9.44 ± 4.82^a^	16.75 ± 3.17^bcd^	0.06 ± 0.25^a^	1.06 ± 1.06^ab^	0.19 ± 0.40^a^	5.94 ± 3.97^cd^
NCU5	9.13 ± 5.33^a^	6.75 ± 2.67^a^	0.00 ± 0.00^a^	0.00 ± 0.00^a^	0.00 ± 0.00^a^	1.00 ± 1.51^a^
NCU6	13.69 ± 5.13^abc^	13.75 ± 5.63^bcd^	0.06 ± 0.25^a^	0.13 ± 0.34^a^	0.00 ± 0.00^a^	2.50 ± 1.90^ab^
NCU7	13.19 ± 5.59^abc^	13.31 ± 3.93^b^	0.13 ± 0.34^a^	0.00 ± 0.00^a^	0.00 ± 0.00^a^	0.13 ± 0.50^a^
NCU8	13.81 ± 5.18^abc^	19.19 ± 5.46^d^	0.06 ± 0.25^a^	0.00 ± 0.00^a^	0.00 ± 0.00^a^	1.50 ± 1.86^a^
NCU9	17.88 ± 6.18^c^	14.88 ± 7.78^bcd^	7.75 ± 4.89^b^	0.31 ± 0.48^ab^	0.00 ± 0.00^a^	0.00 ± 0.00^a^
NCU10	16.69 ± 4.78^bc^	16.81 ± 6.44^bcd^	2.06 ± 1.81^a^	0.88 ± 1.96^ab^	0.00 ± 0.00^a^	0.00 ± 0.00^a^
NGV1	17.19 ± 6.86^c^	17.94 ± 5.94^cd^	8.75 ± 6.18^b^	0.31 ± 0.60^ab^	1.19 ± 1.22^b^	0.00 ± 0.00^a^
NGV2	12.06 ± 4.27^abc^	14.75 ± 4.64^bcd^	9.50 ± 3.74^bc^	1.31 ± 1.49^b^	2.56 ± 2.31^c^	0.00 ± 0.00^a^
NGV3	14.44 ± 5.53^abc^	12.06 ± 3.17^ab^	12.31 ± 6.72^c^	0.63 ± 0.96^ab^	0.63 ± 0.72^ab^	0.00 ± 0.00^a^

^a^ Values followed by the same letter within each column are not significantly different at P<0.05.

### Population’s genetic diversity, structure, and relatedness

In contrast with the race-F_CU_ gene pool (populations CU05 and CU08), with null intrapopulation diversity, some of the new populations from Cuenca exhibited large intrapopulation genetic diversity. Thus, Shannon’s diversity index (I) was particularly high for populations NCU2 (I=0.59), NCU7 (I=0.44), NCU8 (I=0.41), NCU4 (I=0.29), and NCU6 (I=0.24). These values were like those found in the race-G populations from the Guadalquivir Valley, with I=0.31 to 0.45 (**Table 3**). Three new populations from Cuenca (NCU1, NCU3, NCU10) showed null genetic diversity, whereas two populations, NCU5 and NCU9, had intermediate values of I=0.06 and 0.11, respectively (**Table 3**).

**Table 3:** Intrapopulation diversity parameters for populations NCU1 to NCU10 from Cuenca, and control populations SE10, CO02 (race F from the Guadalquivir Valley), CU05, CU08 (race F from Cuenca), and NGV1 to NGV3 (race G from the Guadalquivir Valley): percentage of polymorphic loci (P), observed heterozygosity (Ho), expected heterozygosity (He), and Shannon’s diversity index (I).

Population	P	H_0_ ( ± SE)	He ( ± SE)	I ( ± SE)
NCU1	1,49	0.00 ± 0.00	0.00 ± 0.00	0.00 ± 0.00
NCU2	85,07	0.08 ± 0.00	0.42 ± 0.02	0.59 ± 0.03
NCU3	1,49	0.00 ± 0.00	0.00 ± 0.00	0.00 ± 0.00
NCU4	85,07	0.04 ± 0.00	0.17 ± 0.01	0.29 ± 0.02
NCU5	46,27	0.02 ± 0.00	0.03 ± 0.00	0.06 ± 0.01
NCU6	85,07	0.01 ± 0.01	0.13 ± 0.01	0.24 ± 0.01
NCU7	85,07	0.01 ± 0.00	0.28 ± 0.02	0.44 ± 0.02
NCU8	85,07	0.19 ± 0.01	0.26 ± 0.02	0.41 ± 0.02
NCU9	67,16	0.05 ± 0.01	0.05 ± 0.01	0.11 ± 0.01
NCU10	0,00	0.00 ± 0.00	0.00 ± 0.00	0.00 ± 0.00
SE10	2,99	0.00 ± 0.00	0.00 ± 0.00	0.01 ± 0.00
CO02	0,00	0.00 ± 0.00	0.00 ± 0.00	0.00 ± 0.00
CU05	0,00	0.00 ± 0.00	0.00 ± 0.00	0.00 ± 0.00
CU08	0,00	0.00 ± 0.00	0.00 ± 0.00	0.00 ± 0.00
NGV1	76,12	0.09 ± 0.01	0.31 ± 0.02	0.45 ± 0.03
NGV2	83,58	0.04± 0.01	0.19 ± 0.02	0.31 ± 0.03
NGV3	83,58	0.12 ± 0.01	0.23 ± 0.02	0.35 ± 0.03

Nei’s unbiased genetic identity analysis revealed that nine out of the ten new populations of Cuenca were coincident or very close (identity >0.95) to either the classical gene pool F_CU_, represented by populations CU05 and CU08, or the classical gene pool F_GV_, represented by populations SE10 and CO02. Thus, populations NCU1, NCU3, NCU4, and NCU8 were very close to the F_CU_ gene pool, whereas populations NCU5, NCU6, NCU7, NCU9, and NCU10 were very close to the F_GV_ gene pool (**Table 4**). Only population NCU2 showed identity values lower than 0.95 with both gene pools, 0.74 with F_GV,_ and 0.79 with F_CU_.

**Table 4:** Nei’s unbiased genetic identity values between the new sunflower broomrape populations from Cuenca and between them and the control populations SE10, CO02 (race F from the Guadalquivir Valley), CU05, CU08 (race F from Cuenca), and NGV1 to NGV3 (race G from the Guadalquivir Valley).

	NCU1	NCU2	NCU3	NCU4	NCU5	NCU6	NCU7	NCU8	NCU9	NCU10
NCU2	0.79									
NCU3	1.00	0.79								
NCU4	1.00	0.86	1.00							
NCU5	0.17	0.75	0.16	>0.28						
NCU6	0.24	0.80	0.24	0.35	1.00					
NCU7	0.39	0.89	0.39	0.50	0.97	0.99				
NCU8	0.97	0.91	0.97	0.99	0.39	0.45	0.60			
NCU9	0.18	0.76	0.18	0.30	1.00	1.00	0.98	0.40		
NCU10	0.15	0.74	0.15	0.27	1.00	1.00	0.97	0.38	1.00	
SE10	0.15	0.74	0.15	0.27	1.00	1.00	0.97	0.38	1.00	1.00
CO02	0.15	0.74	0.15	0.27	1.00	1.00	0.97	0.38	1.00	1.00
CU05	1.00	0.79	1.00	1.00	0.16	0.24	0.39	0.97	0.18	0.15
CU08	1.00	0.79	1.00	1.00	0.16	0.24	0.39	0.97	0.18	0.15
NGV1	0.50	0.87	0.50	0.59	0.82	0.85	0.90	0.65	0.84	0.82
NGV2	0.33	0.82	0.33	0.43	0.93	0.95	0.96	0.53	0.94	0.93
NGV3	0.36	0.82	0.36	0.46	0.90	0.92	0.93	0.54	0.91	0.90

**Figure 1:**
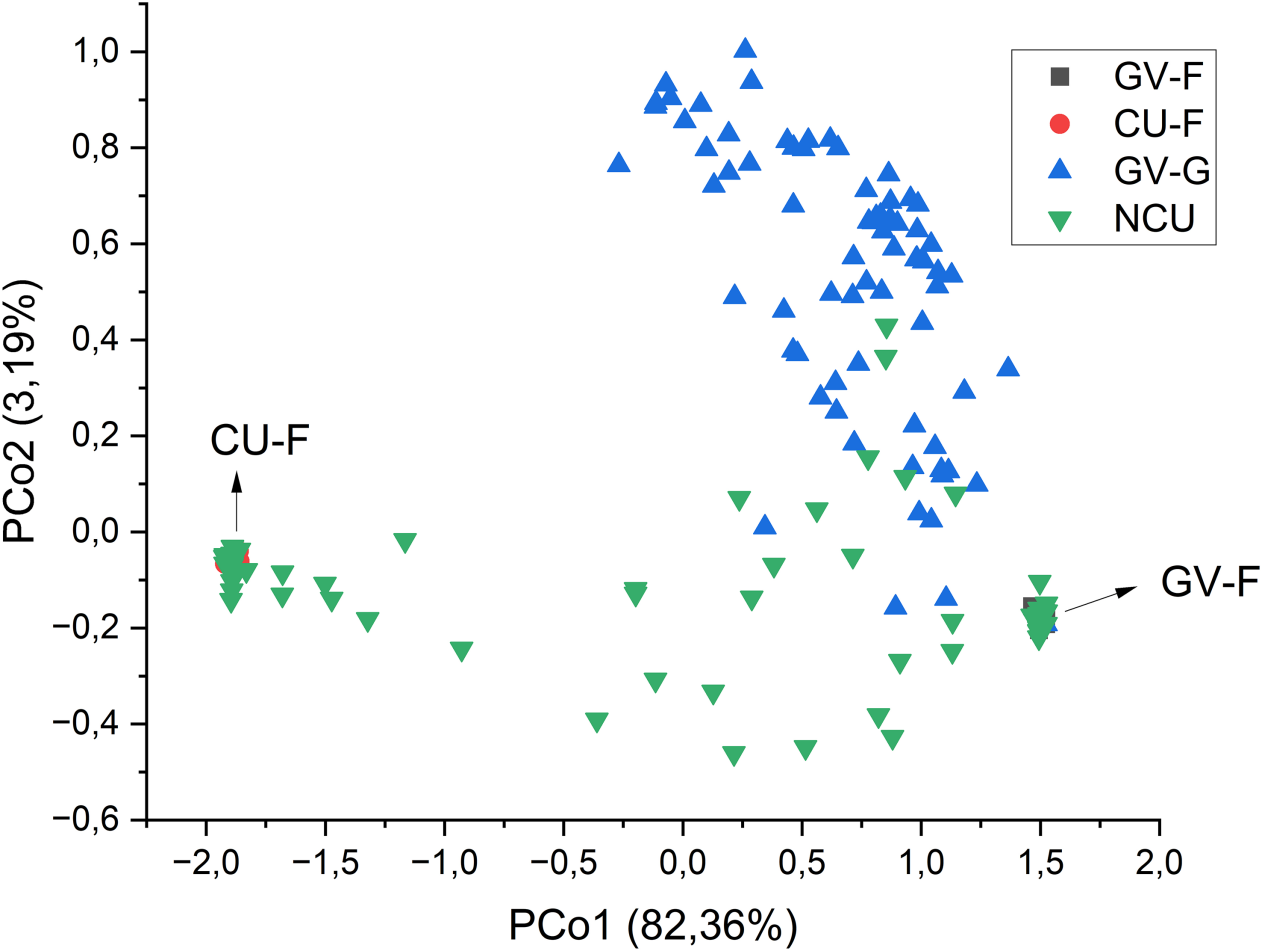
Principal coordinates analysis of the groups of populations from the gene pools of race F of the Guadalquivir Valley (GV-F) and Cuenca (CU-F) and the new populations of both areas (NGV and NCU, respectively). The percentage of variation explained by each principal coordinate is indicated in the axis titles.

To better understand how the new populations from Cuenca relate to the two classical gene pools in Spain and to the new populations with race G_GV_ virulence, the results of the PcoA were used to represent the four groups of populations (races F_GV_, G_GV_, F_CU_, and new populations from Cuenca). The first principal coordinate accounted for 82.36% of the total variation, whereas the second included 3.19%. **Figure 1** shows the biplot of both coordinates. The individuals from the new populations of Cuenca that did not fall in the two traditional gene pools of race F were mainly distributed between both gene pools, with some individuals overlapping with the new populations of the Guadalquivir Valley.

**Figure 2:**
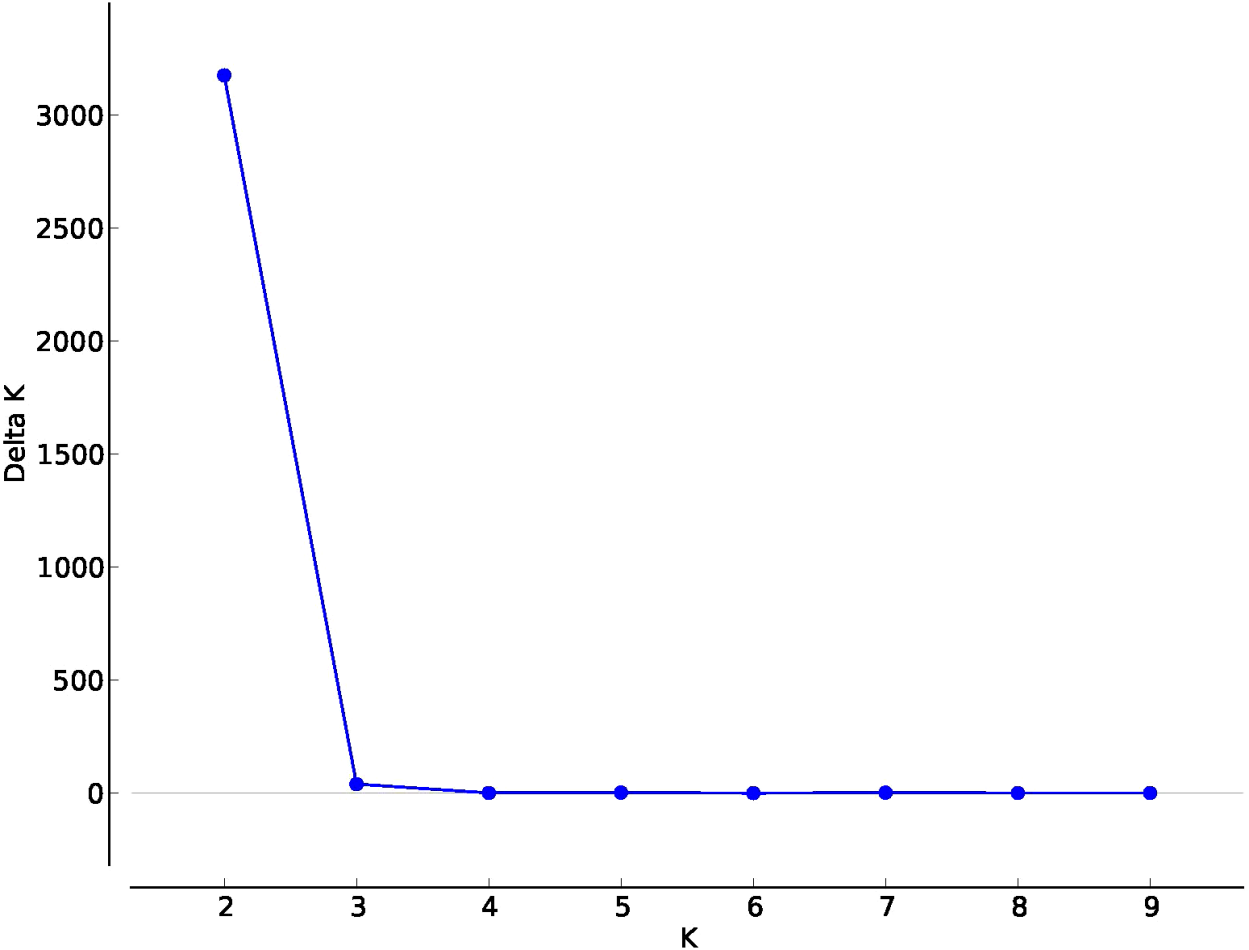
Delta K for values of K in Admixture analysis of genetic structure in a set of populations of sunflower broomrape from Spain.

**Figure 3:**
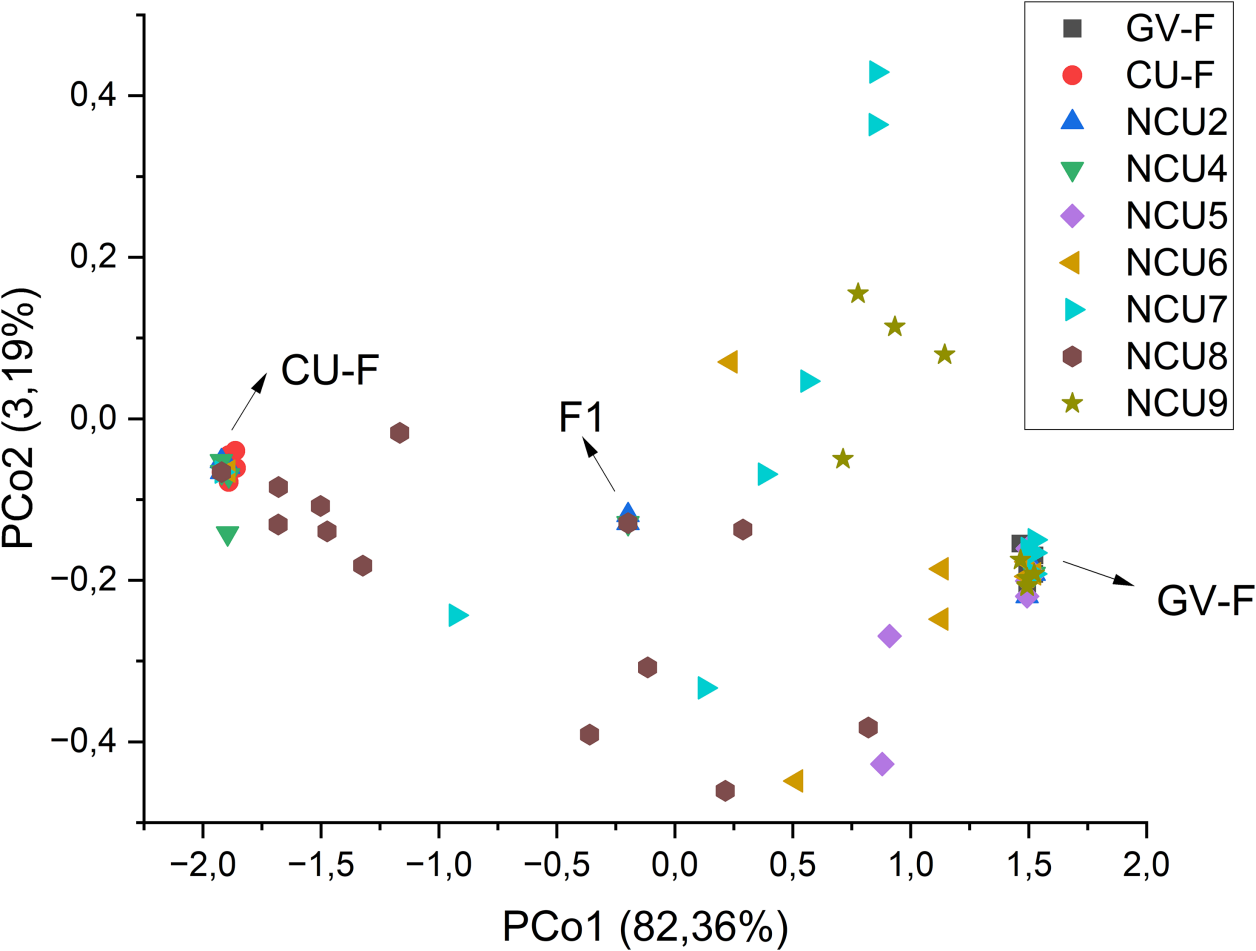
Principal coordinates analysis of the groups of populations from the gene pools of race F of the Guadalquivir Valley (GV-F) and Cuenca (CU-F) and the new populations of Cuenca (NCU), excepting those with null intrapopulation diversity (NCU-1 and NCU-3) that fully belong to the CU-F gene pool. F_1_ indicates putatively hybrid individuals, i.e., those with a percentage of membership to both groups between 49.90% and 50.10% based on population genetic structure analysis. The percentage of variation explained by each principal coordinate is indicated in the axis titles.

Structure analysis was conducted to confirm these results and accurately determine the percentage of the membership of the individuals of the new populations from Cuenca to the classical Spanish gene pools of sunflower broomrape. As expected, the study identified the existence of two genetic groups in the set of populations (**Figure 2**). The analysis of the percentage of membership to each group enabled to distinguish the individuals with full membership in one of the gene pools (>99%), and even to identify F1 individuals (membership to both groups between 49.90% and 50.10%). Thus, this analysis revealed that all the individuals of populations NCU1 and NCU3 had full membership in the gene pool of Cuenca (Group 1). In contrast, all the individuals of population NCU10 had full membership in the gene pool of the Guadalquivir Valley (**Table 5**). Coincident with Nei’s unbiased genetic identity analysis results, populations NCU4 and NCU8 were closer to the Cuenca gene pool, and populations NCU5, NCU6, NCU7, and NCU9 were closer to the Guadalquivir Valley gene pool. Populations NCU2, NCU4, and NCU8 contained putative F_1_ plants, whereas populations NCU5, NCU6, NCU7, NCU8, and NCU9 contained individuals with intermediate membership to the two groups. The PcoA distribution of the populations that did not have full identity with one of the Spanish gene pools, i.e., NCU2 and NCU4 to NCU9, together with the populations of the two original gene pools, is shown in **Figure 3**.

**Table 5:** Results of the genetic structure analysis, indicating the membership to group 1 (G1) and G2, the number of individuals with more than 99% of the membership in G1 and G2, the number of putative F1 individuals (percentage of membership to each group between 49.90% and 50.10%), and rest of individuals with intermediate membership values.

Population	Mean G1 (%)	Mean G2 (%)	G1 (>99%)	G2 (>99%)	F_1_ (50%)	Other
NCU1	99.88	0.12	40	0	0	0
NCU2	52.37	47.63	10	9	2	0
NCU3	99.87	0.13	24	0	0	0
NCU4	93.09	6.91	20	1	1	0
NCU5	1.54	98.46	0	22	0	2
NCU6	8.35	91.65	1	18	0	4
NCU7	21.28	78.73	3	15	0	6
NCU8	79.80	20.20	11	0	2	11
NCU9	3.16	96.84	0	19	0	4
NCU10	0.10	99.90	0	24	0	0
SE10	0.22	99.78	0	12	0	0
CO02	0.10	99.90	0	12	0	0
CU05	99.90	0.10	12	0	0	0
CU08	99.90	0.10	12	0	0	0
NGV1	32.57	67.43	0	0	0	24
NGV2	16.54	83.46	0	2	0	22
NGV3	20.59	79.41	0	0	0	24

## Discussion

Two gene pools of sunflower broomrape have been traditionally present in Spain, one in the Guadalquivir area of southern Spain and another in the Cuenca province in central Spain. The two gene pools are genetically distant and have shallow intrapopulation genetic diversity caused by a founder effect (Pineda-Martos et al., 2013). In that study, the introduction of populations from the Guadalquivir Valley into Cuenca and vice versa was detected for the first time. This fact has explained the appearance of a new race G in the Guadalquivir Valley, putatively caused by the genetic recombination of avirulence loci of both gene pools (Martín-Sanz et al., 2016). After detecting a new virulence of sunflower broomrape in Cuenca province, we investigated whether genetic recombination of avirulence genes could also be at the bottom of this new virulence. Notably, the new virulence observed in Cuenca is mainly characterized by the parasitization of the DEB2 sunflower line, which is resistant to all race-G populations evaluated so far (Martín-Sanz et al., 2016). Based on the results of the present study, the hypothesis of the genetic recombination of avirulence genes present must be discarded as the cause of DEB2 parasitization since the population with the highest degree of attack on DEB2, NCU1, showed full membership to the Cuenca gene pool, with no mixture with any external population. Accordingly, under no evidence of genetic recombination associated with the change in virulence, it can be hypothesized that a point mutation may have caused the virulence change in the Cuenca gene pool. A point mutation was also hypothesized as the genetic mechanism underlying the change from race E to race F virulence in the Guadalquivir Valley since the increased virulence was associated with dominant alleles at a single avirulence locus (Rodríguez-Ojeda et al., 2013) and no changes in population structure and diversity were observed between race-E and race-F populations (Pineda-Martos et al., 2013).

Although genetic recombination seems not to be the cause for DEB2 parasitization, the results of this study showed that genetic recombination between local populations and populations introduced from the Guadalquivir Valley is occurring in Cuenca nowadays. This is more clearly seen in populations NCU2, NCU4, and NCU8, where F_1_ individuals between the Cuenca gene pool plants and the Guadalquivir Valley gene pool have been identified. The case of NCU2 population deserves additional discussion. From 21 individuals analysed, 10 belonged to the Cuenca gene pool, 9 belonged to the Guadalquivir Valley gene pool, and two plants were F_1_. This suggests that the introduction of plants from the Guadalquivir Valley into this population has been very recent, and genetic recombination is at the earliest stage. The case of population NCU10, where all the individuals belong to the Guadalquivir Valley gene pool, suggests that broomrape is being introduced from the Guadalquivir Valley even in areas where populations of the Cuenca gene pool were not present. Broomrape seeds are mainly dispersed through agricultural machinery and tools and together with the host seeds (Habimana et al., 2014). Although how and when the broomrape seeds of the Guadalquivir Valley gene pool have reached Cuenca province cannot be ascertained, it is important to note that populations of this gene pool have been also found in distant places such as northern Spain (Malek et al., 2017) and Morocco (Nabloussi et al., 2023).

This is the first time that complex sunflower broomrape populations have been characterized in detail. Their individuals could be unequivocally classified according to the gene pool of provenance, including identifying F_1_ hybrid plants. This opens the possibility of extending the methodology used in this study to other areas, assuming that identifying the original gene pools is still possible. What we could not separate in the present study were the individuals of the Cuenca gene pool with classical race-F virulence, not attacking DEB2 sunflower population, from those with increased virulence that parasitize on DEB2. Assuming the point mutation hypothesis exposed above, separating both groups would require the availability of markers closely linked to the avirulence locus, which are not currently available.

The nomenclature for sunflower broomrape races has been traditionally based on the use of letters, with A indicating the initial population attacking sunflowers in Russia at the end of the nineteenth century, B the population that overcame resistance to race A, and so on (Cvejić et al., 2020). This led to using the same letter to designate the virulence of populations in different geographic areas with different virulence profiles, which caused great confusion. For that reason, Martín-Sanz et al. (2016) proposed adding a subscript indicating the geographical area of the population. In this case, considering that the most virulent race in the area so far was race F, the new populations attacking DEB2 sunflower line should be named race G. However, it must be considered that race G is being used in other areas to designate populations that overcome the resistance of race-F-resistant hybrids (Škorić et al., 2021), which is the case of population NCU9 but not the other populations. Consistent with this nomenclature, we propose to designate race G_CU_ only to the populations that show a number of shoots significantly higher than zero on Kiara, and race F^+^_CU_ to the populations that show a number of shoots significantly higher than zero on DEB2 but not on Kiara. Any other population parasitizing on NR5 but not parasitizing significantly on Kiara or DEB2 should be considered race F_CU_. Accordingly, NCU5, NCU7, NCU8, and NCU10 are currently race F_CU_, NCU1 to NCU4 and NCU6 are race F^+^_CU_, and population NCU9 is race G_CU_. This is the current situation, although some populations already show a low, non-significant number of shoots on Kiara and/or DEB2 and probably will evolve soon to higher levels of virulence. Thus, it cannot be discarded that some populations, such as NCU3, become virulent on race-F resistant hybrids in addition to their virulence on DEB2. In that case, we propose that they are classified as (F^+^G)_CU_. Concerning race G_CU_ (population NCU9), it can be argued that it was probably introduced from the Guadalquivir Valley and could be considered a race G_GV_. However, with the information available, we cannot discard that this population might have evolved locally in a similar way as populations G_GV_ evolved in the Guadalquivir Valley, as hybridization between both gene pools in Cuenca has been documented in the present research as it was documented previously in the Guadalquivir Valley (Pineda-Martos et al., 2013).

It is generally accepted that resistance to broomrape in sunflower is cumulative, i.e., resistance to a new race also provides resistance to the previous ones (Fernández-Martínez et al., 2015). In this study, we show that sunflower germplasm can be resistant to one race but not to another one, e.g., DEB2 is resistant to G_CU_ but not to F+_CU_, whereas Kiara shows the opposite response. This observation emphasizes the need for a detailed characterization of the existing races of sunflower broomrape and the genetic structure of the populations to have a better picture of how the populations are evolving and the mechanisms driving race evolution. Also, our results highlight the importance of pyramiding resistance genes as a strategy to develop durable resistance to sunflower broomrape. Pyramiding strategies should include genes with a major effect, such as *Or_7_* (Duriez et al., 2019) and *Or_Deb2_* (Fernández-Aparicio et al., 2022), but also genes determining partial or posthaustorial resistance (Martín-Sanz et al., 2020), or even genes involved in polygenic resistance (Pérez-Vich et al., 2013; Louarn et al., 2016; Imerovski et al., 2019).

The race evolution of sunflower broomrape is primarily driven by mutations, consistent with the gene-for-gene host-parasite interaction between this parasite and the sunflower crop (Rodríguez-Ojeda et al., 2013; Škorić et al., 2021). Genetic recombination between avirulence loci has also been proposed as an additional mechanism for race evolution in this species (Martín-Sanz et al., 2016). In our present research, we identified sunflower broomrape populations in which both genetic mechanisms have modulated virulence. These findings provide valuable insights into the mechanisms of race evolution in sunflower broomrape and can aid in developing effective control strategies.

## Data availability statement

The original contributions presented in the study are included in the article/**Supplementary Material**, further inquiries can be directed to the corresponding author.

## Author contributions

LV, AM-S, and BP-V conceived the work and planned and supervised the research. LV and AM-S collected the broomrape populations and evaluated virulence. BF-M, LM, and BP-V carried out plant genotyping. BF-M, BP-V, and LV performed data analyses. BF-M and LV wrote the draft of the manuscript. All authors contributed to the article and approved the submitted version.
